# Data on the movement behaviour of four species of grassland butterfly

**DOI:** 10.1016/j.dib.2019.104611

**Published:** 2019-10-04

**Authors:** Luke C. Evans, Ian Sims, Richard M. Sibly, Pernille Thorbek, Tom H. Oliver, Richard J. Walters

**Affiliations:** a School of Biological Sciences, University of Reading, Whiteknights, PO Box 217, Reading, Berkshire, RG6 6AH, UK; b Syngenta, Jealott's Hill International Research Centre, Bracknell, Berkshire, RG42 6EY, UK; c BASF SE, APD/EE, Speyerer Strasse 2, 67117, Limburgerhof, Germany; d Centre for Environmental and Climate Research, University of Lund, Sweden

**Keywords:** Movement movement-ecology random-walk behaviour Lepidoptera

## Abstract

This Data in Brief article describes data on the movement behaviour of four species of grassland butterflies collected over three years and at four sites in southern England. The datasets consist of the movement tracks of *Maniola jurtina, Aricia agestis, Pyronia tithonus,* and *Melanargia galathea,* recorded using standard methods and presented as steps distances and turning angles. Sites consisted of nectar-rich field margins, meadows, and mown short turf grasslands with minimal flowers. In total, 783 unique movement tracks were collected. The data were used for analysing the movement behaviour of the species and for parameterising individual-based movement models.

**Table:** Specifications Table

Subject	Ecology
Specific subject area	Movement ecology, butterflies
Type of data	Table
How data were acquired	Butterfly positions were recorded with a GNSS receiver (Arrow 200 RTK) with accuracy < 30 cm. Behaviours were timed on a custom Android phone app. Meteorological variables were recorded from weather stations < 3km from the sites and sunshine was recorded using data loggers deployed at sites (HOBO pendant).
Data format	Processed.
Parameters for data collection	Data on butterfly movements were collected in the summers of 2016–2018 at four sites in southern England. Observations took place primarily between 10:00 am and 16:00 pm and in dry conditions, though air temperature and cloud cover varied widely.
Description of data collection	Butterflies were observed in the field with the positions of individuals recorded by placing a numbered marker flag, either at each landing site or after every 15 seconds during a continuous flight [3,4]. The precise location of each flag was then mapped using a Global Navigation Satellite System receiver (Arrow 200 RTK). Flight duration and behaviour were recorded on a bespoke Android App developed for the project. Records of location, time and behaviour were processed to calculate the distance between successive flags as step distances and the angle subtended between consecutive flags as turning angles.
Data source location	North farm Oxfordshire 51°37′N, 1°09′W Jealott's Hill farm Berkshire 51°27′N, 0°44′W University of Reading Berkshire 51.4414° N, 0.9418° W Sonning farm Berkshire 51°28′N, 0°53′W
Data accessibility	Mendeley data [6] Published: 30 Aug 2019|Version 1|https://doi.org/10.17632/kpcgkfmpv8.1https://data.mendeley.com/datasets/kpcgkfmpv8/1
Related research article	Author's names: Luke C. Evans, Richard M. Sibly, Pernille Thorbek, Ian Sims, Tom H. Oliver and Richard J. Walters Title: Quantifying the effectiveness of agri-environment schemes for a grassland butterfly using individual-based models. Journal: Ecological modelling *411*, 108798. DOI: 10.1016/J.ECOLMODEL.2019.108798

**Table:** 

**Value of the Data** • The data consist of a large number of high-accuracy movement and behavioural observations of four species of grassland butterfly. Movement was recorded across a range of weather conditions and at sites of varying in resource density. The data are useful for analyses of the behaviour and/or movement of butterflies. • The dataset is particularly useful for understanding how the movement of butterflies is affected by varying weather conditions or habitat quality. • The data can be used to parameterise random-walk movement models as movement was recorded as step distances and turning angles using standard methods.

## 1 Data

The dataset consists of movement and behavioural observations of four species of grassland butterfly measured at four sites in southern England. The data files were deposited in Mendeley data (https://doi.org/10.17632/kpcgkfmpv8.1) and consist of two tables representing 783 unique movement tracks. Data collected in 2016 and 2017 are supplementary to [1] and data from 2017 and 2018 to [2].

## 2 Experimental design, materials, and methods

### 2.1 Study sites

The study was conducted at four sites in southern England over the summers of 2016, 2017 and 2018. This sites were North farm in Oxfordshire (51°37′N, 1°09′W), Jealott's Hill farm Berkshire (51°27′N, 0°44′W), the University of Reading Berkshire (51.4414° N, 0.9418° W), and Sonning farm Berkshire (51°28′N, 0°53′W). The farm sites were agricultural areas where agri-environment schemes had been implemented and consisted of a mixture of arable fields, open meadows, and nectar-rich field margins. The sites at the University were selected as a comparison between flower-rich and flower-poor, two areas were mown short turf grasslands with minimal flowering plants and the other areas were meadow grasslands containing a variety of grass species and wildflowers predominantly the common knapweed (*Centaurea nigra* Linnaeus).

### 2.2 Materials and methods

Individual butterflies were recorded opportunistically in the field between the hours of 10:00 and 16:00. Butterflies were followed at a distance from the recorder of approximately 3 m for up to 10 minutes. During this period, the position of each individual was recorded by planting a sequentially numbered marker flag, either at each landing site or after every 15 seconds during continuous flight, following established methodology [4,5]. The precise location of each flag was then mapped using a high-grade Global Navigation Satellite System receiver (Arrow 200 RTK) accurate to < 30cm. Observations were stopped early either if the butterfly could no longer be tracked (i.e. crossed hedges or lost from sight) or if a maximum number flags were used. A maximum of 20 flags was used in 2016 and 2017, and 15 flags in 2018. During the observations activity was recorded continuously by categorising behaviour into flying, nectaring (taking nectar from flowers), basking (open wings and stationary), inactive (closed wing and stationary), and oviposition [5].

Dataloggers (HOBO pendant) were used to record solar radiation (lux) at 10-s intervals and the air temperature was measured at hourly intervals from meteorological stations within 3km the sites (Jealotts Hill, Sonning, University of Reading, RAF Benson). The dataloggers measure a broad spectrum of light wavelengths and are most effective at measuring the relative light intensity.

### 2.3 Data processing

Records of precise location, time and behaviour were processed to calculate the distance between successive flags, referred to as a step distances, and the angle subtended between consecutive flags, referred to as turning angles. The data is collated into two tables. The first, from the summer of 2016, is organised such that each row refers to a unique flag with step distance to the flag, the turning angle and the amount of time the butterfly remained stationary recorded on a single row. Behaviours performed by the butterfly were then recorded together in a single cell. In the second dataset, from the summers of 2017 and 2018, step distances and turning angles were as in 2016, but each row refers to a separate behaviour so that multiple rows record behaviours at the same position. This allowed a simpler analysis of the sequence and duration of behaviours. In addition, both datasets contain information on the year, day of the year, species, sex, solar radiation, air temperature, location and site quality (nectar-poor or nectar-rich) of an observation.
